# Editorial: Game Changers in Inflammatory Bowel Diseases

**DOI:** 10.3389/fmed.2021.795597

**Published:** 2021-11-18

**Authors:** Anita Bálint, Barbara Dorottya Lovász

**Affiliations:** ^1^Department of Medicine, University of Szeged, Szeged, Hungary; ^2^Faculty of Health Sciences, Institute of Applied Health Sciences, Semmelweis University, Budapest, Hungary

**Keywords:** inflammatory bowel disease, Crohn's disease, ulcerative colitis, biological therapies, biomarkers, microbiome, new advanced technologies

## Background

The accurate etiology of inflammatory bowel disease (IBD)—[Crohn's disease (CD) and ulcerative colitis (UC)] is still unknown. According to the accepted theory, the disease seems to be the consequence of an abnormal immune response induced by luminal antigen exposure. In the absence of clear etiology, there is no targeted treatment that could be cure IBD.

Moreover, the heterogeneity and complexity of UC and CD greatly complicate their treatment. The disease dramatically decreases the patients' quality of life and accounts for substantial costs to the healthcare system and society. Currently, the therapeutic goal includes not only clinical and endoscopic remission but also mucosal healing, i.e., deep remission, therefore basic and clinical research are both needed.

Considering these facts, the significance of the scientific research and innovations of the last decades, which have enriched the therapeutic tools of the gastroenterologist, are evident. Numerous studies have been published on new management lines, innovative drugs, serum, and fecal markers. Are the introduction of new molecules as anti-integrin antibodies, anti-interleukin (IL)-23 antibodies, Janus Kinase (JAK) inhibitors, or mesenchymal stem cells game changers? Or is the application of right predictors and excellent monitoring the key? How could we improve patient management? What new opportunities do we have?

## Etiopathology of IBD: Focus on Gut Microbiome

Thanks to the next-generation technologies (including 16S rRNA, 18S rRNA, internal transcribed spacer sequencing, shotgun metagenomic sequencing, metatranscriptomic sequencing, and viromic sequencing), the connection of gut microbiome, and a wide range of diseases was confirmed. The more associations we explore, the more the function encoded in the intestinal microbiome appears to be significant. Microbiome analysis software tools are constantly evolving. While amplicon-based sequencing (16S rRNA, 18S rRNA) methods usually target a single gene, shotgun metagenomic sequencing adds a detailed layer to the taxonomic characterization of the microbial community by providing information on the gene composition and the functional capacity of the gut microbiome. Moreover, metatranscriptomic sequencing allows researchers to identify the activity of the microbe, in parallel activity of gene expression. Although it is a very promising tool, it must be taken into account that sometimes multiple metagenomic analysis methods may produce inconsistent results even if the same databases are used, therefore standardization of data processing and analysis is warranted (1).

However, several IBD research results have been achieved recently by using these technologies. Individuals with genetic susceptibility when exposed to external triggers are at increased risk at developing IBD. As a summary by Cai et al. shows, the accumulation of potentially pathogenic microorganisms in the gut can be responsible for intestinal inflammation. Moreover, these microorganisms appear to be shared with those of periodontitis and may play a role in the comorbidity of periodontitis and IBD. Changes in the gut microbiome can be responsible for not only the development of IBD, but even for the recurrence after surgical resection. Alteration in gut microbiota can be associated with relapse of CD. These changes are even more important in high-risk patients who need surgical treatment. However, the efficacy of microbial-based therapies in preventing postoperative recurrence of CD is still limited. In the systematic review by Zhuang et al., the mucosa-associated microbiota in surgical biopsy of CD patients is significantly distinct from that in normal mucosa from healthy subjects. Recolonization with recurrence-associated bacteria of gut microbiome after intestinal resection might be associated with post-operative recurrence in CD patients.

Besides dysbacteriosis, long-standing, chronic infections might be another interesting issue in the etiopathology of IBD and even more in the development of complications. Patients with long-standing IBD and cytomegalovirus (CMV) infections are at increased risk of gastrointestinal neoplasms. The connection between them is supported by a case report of a family with an aggressive ATP4A-mutated gastric neuroendocrine tumor (gNET) who had a concurrent inflammatory disease. The progression of ileitis-associated neoplasia was compatible with IBD and CD. Results described by Calvete et al. are important since they contribute to describing the genetic landscape of these clinical associations: inflammation in the intestine was observed as a primary disease and not derived from the gastric neoplasm. Therefore, the findings of this report are directing the attention of gastroenterologists to monitor patients with inflammatory diseases that may trigger other severe pathologies.

## Diagnostic Tools: What's New in the Field of Endoscopy?

Endoscopy plays a defining role in the diagnosis and management of IBD, additionally, it is fundamental to determine one of our therapeutic goal, the deep remission. We need more and more precise methods to identify inflammation and other pathologies on gut mucosa as prevention becomes the focus of patient management. Recently, most of advances of endoscopy aims to get as close as possible to microscopic findings. New diagnostic methods have been developed, such as dye-less chromoendoscopy (narrow band imaging, Fuji intelligent color enhancement, i-scan, blue light imaging, linked color imaging, etc.), endocytoscopy–endomicroscopy, and molecular imaging, which is based on the topical or intravenous administration of specific label substance and the subsequent visualization of targeted structures through simultaneous confocal imaging. The new pan-enteric video capsules with latest technologies provide more effective inflammation detection. The benefits of artificial intelligence system are reduction of operator-subjectivity and inter-observer variability (2). In the near future, artificial intelligence can be a useful tool in the real-time assistance of doctors' diagnostic decisions during endoscopy in everyday practice. Deep learning algorithms have a high accuracy in the detection of CD ulcers with video capsule endoscopy. This could be a practical application for example in the differential diagnosis of CD and non-steroidal anti-inflammatory drugs (NSAID) ulcers. Non-steroidal anti-inflammatory drugs-induced ulcers are one of the most important differential diagnosis for small bowel ulcers in patients evaluated for suspected CD. A deep learning network was trained by Klang et al., using Crohn's ulcer images from capsule endoscopy and evaluated its performance for NSAID ulcers.

## Therapeutic Advances: New Molecules, Old Molecules…New Combinations

The first drug that reduced IBD mortality by orders of magnitude was the corticosteroid. Thiopurines have been another cornerstone in the treatment of IBD. There is a growing interest in optimizing thiopurine therapy, especially in the era of biological therapies. Gargallo-Puyuelo et al. are reviewing the pros and cons of the efficacy and safety issues of conventional thiopurine therapy and the benefits from personalized therapy with thiopurines.

The introduction of biological agents was a real breakthrough in the management of severe IBD. Nevertheless, treatment non-response, allergy or infusion reactions, and other adverse events limit the use of these drugs. There are several studies that may represent new therapeutic options, like anti-integrin antibodies (vedolizumab, etrolizumab, AJM300, ontamalimab), blockade of interleukin-23 (ustekinumab, briakinumab, brazikumab, risankinumab, mirikizumab, guselkumab), JAK inhibitors (upadacitinib, filgotinib), modulation of sphingosine-1-phosphate receptors (fingolimod, ozanimod, etrasimod, amiselimod), phosphodiesterase inhibitors (apremilast), oligonucleotide-based drugs (morgensen, alicaforsen, cobitolimod), stem cell therapy, and fecal microbiota transplantation (3). Promising research is under process related to the efficacy and safety of V565-oral anti-TNF therapy (4).

The choice of treatment depends on the phenotype, activity, and complications of the IBD. To note, several factors play a role in the effectiveness of therapy, even the doctors themselves, as well as their preferences. Abdullah et al. evaluated the adherence of gastroenterologists at an IBD center, to anti-TNF (anti-tumor necrosis factor) combination therapy. Tight disease control, the timing of biological therapy, and monitoring of serum antibody levels can be affected by different groups of gastroenterologists according to the age and interest in IBD.

## Prognosis: Predictors of Treatment Response and IBD Outcome

Appropriate diagnosis, treatment decision, and monitoring can have a significant impact on beneficial IBD outcomes. What can we do? The sole predictor or biomarker, which is sensitive and specific enough to be used alone in the prognosis and the outcome of IBD does not exist, but we have some useful, low-cost, and reliable tools like C-reactive protein (CRP), antibodies (pANCA: perinuclear anti-neutrophil cytoplasmic antibodies, ASCA: Anti-*Saccharomyces cerevisiae* antibodies), and fecal calprotectin. Although there have been some promising results from studies, some biomarkers suitability is questionable and requires further investigation: LL-37 (serum cathelicidin), TFF3 (trefoil factor 3), cytokines (IL-6, TNF, etc.), fibrinogen, anti-outer membrane protein, antibodies to flagellin, anti-12 antibody, anti-carbohydrate antibody, fecal lactoferrin, fecal neopterin, fecal polymorphonuclear neutrophil elastase, fecal S100A12 (5). A lot of research are being carried out about the role of circulating non-coding microRNA besides metabolomics and proteomics studies. Some serum immunoglobulins were associated with specific disease phenotypes of CD. Yang et al. revealed that neuron-specific enolase and CRP could be used as non-invasive tests in detecting the location and severity of disease in patients with CD in daily routine practice. The mucosal healing index [including carcinoembryonic antigen-related cell adhesion molecule, vascular cell adhesion molecule, CRP, serum amyloid A, angiopoietin-1, angiopoietin-2, matrix metalloproteinase (MMP)-1, MMP-2, MMP-3, MMP-9, extracellular MMP inducer, transforming growth factor-α, and IL-7] was created using multiple logistic regression models to predict mucosal healing in patients with CD. The same in UC patients who received anti-TNF therapy was established with CRP, CHI3L1 (anti-chitinase-3-like protein 1), LL-37, and neutrophil count (6). Therapeutic drug monitoring has been suggested to be applied in case of monitoring for dose escalation, de-escalation, or switch treatment (7).

In the treatment of pediatric patients with CD, risk-stratification is mandatory during diagnosis in order to identify high-risk patients who need early intensive therapy to prevent poor outcomes. The consensus guidelines of ESPGHAN/ECCO describe predictors of poor outcome (POPO) as means for risk stratification. De Laffolie et al. investigated therapy stratifying potential comparing POPO-positive and -negative CD patients from CEDATA-GPGE®, a German-Austrian Registry for Pediatric Inflammatory Bowel Disease to describe “POPO” criteria test statistical properties. They found that POPO with complicated courses of disease were common, hence an early intensified management for pediatric CD patients with POPO-positivity should be considered.

Unfortunately, varying degrees of disability are common in IBD. This is due to different underlining mechanisms, but certain prognostic factors could help to identify the group of patients at significantly higher risk. Bian et al. reported that disability is mostly correlated with disease activity as well as BMI.

## Conclusions

A great number of landmark studies have been published on new therapeutic strategies, disease monitoring, and complication management in recent years. It also means that we must constantly educate and train ourselves to provide up-to-date and optimal care to our patients. Due to the multitude of new as well as already established treatments, we have the opportunity to apply patient-tailored therapy that have a great significance in achieving better disease outcome.

## Author Contributions

Both authors listed have made a substantial, direct and intellectual contribution to the work, and approved it for publication.

## Funding

This work was supported by the research grants of the National Research, Development and Innovation Office (Grant Nos. 125377, 129266, and 134863), by the New National Excellence Program of the Ministry of Human Capacities (UNKP-20-5-SZTE-161) and Janos Bolyai Research Grant (BO/00598/19/5), and the Géza Hetényi Research Grant by the Faculty of Medicine, University of Szeged.

## Conflict of Interest

The authors declare that the research was conducted in the absence of any commercial or financial relationships that could be construed as a potential conflict of interest.

## Publisher's Note

All claims expressed in this article are solely those of the authors and do not necessarily represent those of their affiliated organizations, or those of the publisher, the editors and the reviewers. Any product that may be evaluated in this article, or claim that may be made by its manufacturer, is not guaranteed or endorsed by the publisher.
